# “Ossified”

**DOI:** 10.1007/s10912-025-09967-0

**Published:** 2025-07-28

**Authors:** Sara B. Khan

**Affiliations:** Northeast Ohio Medical University, 4209 OH-44, Rootstown, OH 44272 United States

I think I lost something

in the cave of their cheek

between the rivers of muscle

somewhere uncharted

where the probes can’t reach

a part of my living soul

was left in them 

something hardened

something fossilized

in me 

there was ihsan in the tears

I wept for death

as if every cold body had

blood of my own

as if every gray eye had

held their gaze with mine 

as if I once knew them

and loved them 

But now my face is bone-dry. 

I no longer wince with my

proximity to their heart

as I gently reveal their

intimate arteries

Now I am proud of the

nerves I have pulled

and I no longer see their

gaping mouths

their peeking eyes

their outstretched arms

their unveiled hands 

I think I lost something soft

Maybe it was hardened

under the lights 

One day I will face the ones

who knew them and loved them

I will have to pretend like I didn’t

see their father without skin

I will have to pretend like I didn’t smile and

prod their mother’s spinal cord

I will cry and pretend that I always have

and that I never stopped.

And the I never lost something

that is gone.

## Data Availability

No datasets were generated or analysed during the current study.

